# (*E*)-Methyl *N*′-[(1*H*-indol-3-yl)methyl­idene]hydrazinecarboxyl­ate 0.25-hydrate

**DOI:** 10.1107/S1600536811026249

**Published:** 2011-07-09

**Authors:** Lu-Ping Lv, Tie-Ming Yu, Wen-Bo Yu, Xian-Chao Hu

**Affiliations:** a Linjiang College, Hangzhou Vocational and Technical College, Hangzhou 310018, People’s Republic of China; b Research Center of Analysis and Measurement, Zhejiang University of Technology, Hangzhou 310014, People’s Republic of China

## Abstract

The asymmetric unit of the title compound, C_11_H_11_N_3_O_2_·0.25H_2_O, contains two independent organic mol­ecules and a water mol­ecule, which lies on a twofold rotation axis. The side chains of the two mol­ecules have slightly different orientations, the C=N—N—C torsion angle being −163.03 (15)° in one and −177.52 (14)° in the other, with each adopting a *trans* configuration with respect to the C=N bond. In the crystal, mol­ecules are linked into chains extending along *b* by N—H⋯O, O—H⋯N and O—H⋯O hydrogen bonds and in addition, four inter­molecular C—H⋯π inter­actions are present.

## Related literature

For general background to Schiff bases, see: Cimerman *et al.* (1997 ▶); Offe *et al.* (1952 ▶); Richardson *et al.* (1988 ▶). For related structures, see: Shang *et al.* (2007 ▶); Tamboura *et al.* (2009 ▶).
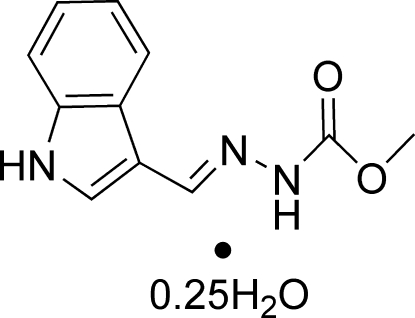


## Experimental

### 

#### Crystal data

C_11_H_11_N_3_O_2_·0.25H_2_O
*M* *_r_* = 886.93
Monoclinic, 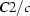

*a* = 27.842 (2) Å
*b* = 11.7574 (11) Å
*c* = 18.565 (2) Å
β = 130.558 (5)°
*V* = 4617.2 (8) Å^3^
*Z* = 16
Mo *K*α radiation
μ = 0.09 mm^−1^
*T* = 223 K
0.21 × 0.17 × 0.15 mm

#### Data collection

Bruker SMART CCD area-detector diffractometer
Absorption correction: multi-scan (*SADABS*; Bruker, 2002 ▶) *T*_min_ = 0.977, *T*_max_ = 0.989
21333 measured reflections
4052 independent reflections
3083 reflections with *I* > 2σ(*I*)
*R*_int_ = 0.029

#### Refinement

*R*[*F* ^2^ > 2σ(*F* ^2^)] = 0.040
*wR*(*F* ^2^) = 0.124
*S* = 1.05
4052 reflections
298 parameters
1 restraint
H atoms treated by a mixture of independent and constrained refinement
Δρ_max_ = 0.16 e Å^−3^
Δρ_min_ = −0.19 e Å^−3^

### 

Data collection: *SMART* (Bruker, 2002 ▶); cell refinement: *SAINT* (Bruker, 2002 ▶); data reduction: *SAINT*; program(s) used to solve structure: *SHELXS97* (Sheldrick, 2008 ▶); program(s) used to refine structure: *SHELXL97* (Sheldrick, 2008 ▶); molecular graphics: *SHELXTL* (Sheldrick, 2008 ▶); software used to prepare material for publication: *SHELXTL*.

## Supplementary Material

Crystal structure: contains datablock(s) I, global. DOI: 10.1107/S1600536811026249/zs2120sup1.cif

Structure factors: contains datablock(s) I. DOI: 10.1107/S1600536811026249/zs2120Isup2.hkl

Supplementary material file. DOI: 10.1107/S1600536811026249/zs2120Isup3.cml

Additional supplementary materials:  crystallographic information; 3D view; checkCIF report

## Figures and Tables

**Table 1:** Hydrogen-bond geometry (Å, °) *Cg*4 and *Cg*5 are the centroids of the C16–C19/N4 and C12–C17 rings, respectively.

*D*—H⋯*A*	*D*—H	H⋯*A*	*D*⋯*A*	*D*—H⋯*A*
N1—H1⋯O3^i^	0.86	2.15	2.9426 (18)	154
O1*W*—H1*WA*⋯N2^ii^	0.93 (2)	2.19 (2)	3.0773 (17)	161 (2)
O1*W*—H1*WA*⋯O1^ii^	0.93 (2)	2.60 (2)	3.2217 (14)	125 (2)
N6—H6⋯O1*W*	0.86	2.17	3.0087 (17)	166
C7—H7⋯*Cg*4^iii^	0.93	2.85	3.594 (3)	137
C9—H9⋯*Cg*5^iii^	0.93	2.74	3.537 (3)	145
C22—H22*A*⋯*Cg*4^iv^	0.96	2.92	3.555 (4)	125
C22—H22*A*⋯*Cg*5^iv^	0.96	2.86	3.796 (3)	125

Symmetry codes: (i) 
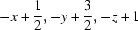
; (ii) 
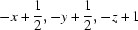
; (iii) 
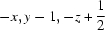
; (iv) 
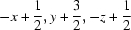
.

**Fig. 1.**
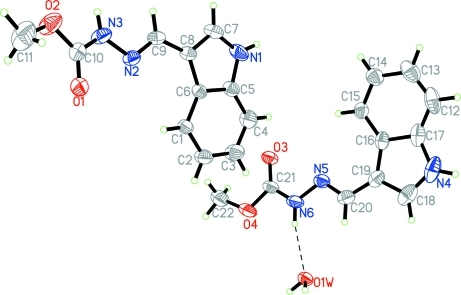
The molecular conformation and atom numbering scheme for the two independent organic molecules and the water molecule of solvation in the asymmetric unit of the title compound. The water molecule lies on a twofold rotation axis and the hydrogen bond is shown as a dashed line. Displacement ellipsoids are drawn at the 30% probability level.

**Fig. 2.**
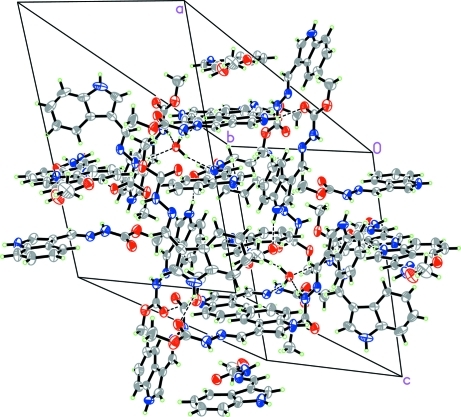
Part of the crystal packing of the title compound in the unit cell. Hydrogen bonds are shown as dashed lines.

## supplementary crystallographic information

### Comment

Schiff bases have attracted much attention due to their potential analytical applications (Cimerman *et al.*, 1997). They are also important ligands, which have been reported to have mild bacteriostatic activity and are used as potential oral iron-chelating drugs for genetic disorders such as thalassemia (Offe *et al.*, 1952; Richardson *et al.*, 1988). Metal complexes based on Schiff bases have received considerable attention because they can be utilized as model compounds of active centres in various complexes (Tamboura *et al.*, 2009). We report here the crystal structure of the title compound C_11_H_11_N_3_O_2_ . 0.25H_2_O.

The title compound (Fig. 1) has two independent, but almost conformationally identical molecules in the asymmetric unit, together with a water molecule of solvation which lies on a twofold rotation axis. Each molecule adopts a *trans* configuration with respect to the C═ N bond. The N2/N3/O1/O2/C10/C11 and N5/N6/O3/O4/C21/C22 planes form dihedral angles of 20.39 (6)° and 16.57 (6)°, respectively, with the C1—C8/N1 and C12—C19/N4 planes. The dihedral angle between the two independent benzene rings is 64.06 (4)°. The bond lengths and angles are comparable to those observed for methyl *N*'-[(*E*)-4-methoxybenzylidene]hydrazinecarboxylate (Shang *et al.*, 2007).

The molecules are linked into chains extending along the *b* axis by N—H···O, O—H···N and O—H···O hydrogen bonds (Table 1 and Fig. 2). In addition, four intermolecular C—H···π interactions are present.

### Experimental

1H-Indole-3-carbaldehyde (1.45 g, 0.01 mol) and methyl hydrazinecarboxylate (0.90g, 0.01 mol) were dissolved in stirred methanol (15 ml) and left for 3.5 h at room temperature. The resulting solid was filtered off and recrystallized from ethanol to give the title compound in 93% yield. Single crystals suitable for X-ray analysis were obtained by slow evaporation of an ethanol solution at room temperature (m.p. 465–467 K).

### Refinement

The water H atom was located in a difference Fourier and both positional and isotropic displacement parameters were refined. Other H atoms were positioned geometrically (N—H =  0.86 Å and C—H = 0.93 or 0.96 Å) and refined using a riding model, with *U*_iso_(H) = 1.2*U*_eq_(C,N) and 1.5*U*_eq_(C_methyl_). A rotating group model was used for the methyl H atoms.

### Crystal data

**Table:**

C_11_H_11_N_3_O_2_·0.25H_2_O	*F*(000) = 1864
*M**_r_* = 886.93	*D*_x_ = 1.276 Mg m^−^^3^
Monoclinic, *C*2/*c*	Melting point = 465–467 K
Hall symbol: -C 2yc	Mo *K*α radiation, λ = 0.71073 Å
*a* = 27.842 (2) Å	Cell parameters from 4052 reflections
*b* = 11.7574 (11) Å	θ = 1.9–25.0°
*c* = 18.565 (2) Å	µ = 0.09 mm^−^^1^
β = 130.558 (5)°	*T* = 223 K
*V* = 4617.2 (8) Å^3^	Block, colourless
*Z* = 16	0.21 × 0.17 × 0.15 mm

### Data collection

**Table:**

Bruker SMART CCD area-detector diffractometer	4052 independent reflections
Radiation source: fine-focus sealed tube	3083 reflections with *I* > 2σ(*I*)
graphite	*R*_int_ = 0.029
φ and ω scans	θ_max_ = 25.0°, θ_min_ = 1.9°
Absorption correction: multi-scan (*SADABS*; Bruker, 2002)	*h* = −33→33
*T*_min_ = 0.977, *T*_max_ = 0.989	*k* = −13→13
21333 measured reflections	*l* = −22→22

### Refinement

**Table:**

Refinement on *F*^2^	Primary atom site location: structure-invariant direct methods
Least-squares matrix: full	Secondary atom site location: difference Fourier map
*R*[*F*^2^ > 2σ(*F*^2^)] = 0.040	Hydrogen site location: inferred from neighbouring sites
*wR*(*F*^2^) = 0.124	H atoms treated by a mixture of independent and constrained refinement
*S* = 1.05	*w* = 1/[σ^2^(*F*_o_^2^) + (0.0709*P*)^2^ + 0.7592*P*] where *P* = (*F*_o_^2^ + 2*F*_c_^2^)/3
4052 reflections	(Δ/σ)_max_ < 0.001
298 parameters	Δρ_max_ = 0.16 e Å^−^^3^
1 restraint	Δρ_min_ = −0.19 e Å^−^^3^

### Special details

**Table:**

Geometry. All esds (except the esd in the dihedral angle between two l.s. planes) are estimated using the full covariance matrix. The cell esds are taken into account individually in the estimation of esds in distances, angles and torsion angles; correlations between esds in cell parameters are only used when they are defined by crystal symmetry. An approximate (isotropic) treatment of cell esds is used for estimating esds involving l.s. planes.
Refinement. Refinement of F^2^ against ALL reflections. The weighted R-factor wR and goodness of fit S are based on F^2^, conventional R-factors R are based on F, with F set to zero for negative F^2^. The threshold expression of F^2^ > 2sigma(F^2^) is used only for calculating R-factors(gt) etc. and is not relevant to the choice of reflections for refinement. R-factors based on F^2^ are statistically about twice as large as those based on F, and R- factors based on ALL data will be even larger.

### Fractional atomic coordinates and isotropic or equivalent isotropic displacement parameters (Å^2^)

**Table:**

	*x*	*y*	*z*	*U*_iso_*/*U*_eq_	
C1	0.37219 (7)	0.37491 (12)	0.56024 (11)	0.0531 (4)	
H1A	0.4010	0.3162	0.5814	0.064*	
C2	0.30987 (8)	0.35174 (15)	0.51235 (14)	0.0696 (5)	
H2	0.2965	0.2765	0.5014	0.083*	
C3	0.26622 (9)	0.43864 (17)	0.47977 (16)	0.0820 (6)	
H3	0.2242	0.4201	0.4476	0.098*	
C4	0.28342 (9)	0.55074 (16)	0.49370 (16)	0.0789 (5)	
H4	0.2539	0.6085	0.4715	0.095*	
C5	0.34667 (8)	0.57480 (12)	0.54227 (13)	0.0584 (4)	
C6	0.39200 (7)	0.48869 (12)	0.57692 (10)	0.0475 (3)	
C7	0.43931 (9)	0.65934 (14)	0.61492 (13)	0.0665 (5)	
H7	0.4696	0.7161	0.6391	0.080*	
C8	0.45160 (7)	0.54508 (13)	0.62345 (11)	0.0522 (4)	
C9	0.51368 (7)	0.50072 (14)	0.67094 (11)	0.0552 (4)	
H9	0.5470	0.5521	0.7009	0.066*	
C10	0.60747 (8)	0.27236 (15)	0.71437 (11)	0.0607 (4)	
C11	0.69308 (11)	0.1737 (2)	0.7468 (2)	0.1228 (10)	
H11A	0.7380	0.1807	0.7843	0.184*	
H11B	0.6730	0.1700	0.6809	0.184*	
H11C	0.6839	0.1056	0.7644	0.184*	
C12	0.10157 (13)	0.8858 (2)	0.10052 (18)	0.0940 (8)	
H12	0.0845	0.9202	0.0432	0.113*	
C13	0.15456 (13)	0.9284 (2)	0.1845 (2)	0.0928 (7)	
H13	0.1743	0.9920	0.1842	0.111*	
C14	0.17960 (9)	0.87807 (16)	0.27089 (15)	0.0760 (5)	
H14	0.2158	0.9085	0.3271	0.091*	
C15	0.15154 (7)	0.78446 (14)	0.27389 (12)	0.0591 (4)	
H15	0.1682	0.7524	0.3318	0.071*	
C16	0.09771 (8)	0.73753 (14)	0.18920 (11)	0.0575 (4)	
C17	0.07411 (10)	0.78978 (18)	0.10343 (13)	0.0741 (5)	
C18	0.01498 (10)	0.6371 (2)	0.06843 (15)	0.0889 (7)	
H18	−0.0161	0.5817	0.0330	0.107*	
C19	0.05859 (8)	0.63973 (15)	0.16554 (12)	0.0629 (4)	
C20	0.06170 (8)	0.56012 (14)	0.22699 (14)	0.0649 (5)	
H20	0.0370	0.4947	0.2013	0.078*	
C21	0.13167 (8)	0.50520 (12)	0.46021 (14)	0.0601 (4)	
C22	0.16560 (12)	0.40801 (19)	0.59737 (16)	0.0963 (7)	
H22A	0.1588	0.3377	0.6159	0.144*	
H22B	0.1545	0.4705	0.6174	0.144*	
H22C	0.2093	0.4140	0.6265	0.144*	
N1	0.37741 (8)	0.67819 (11)	0.56674 (12)	0.0742 (4)	
H1	0.3599	0.7439	0.5534	0.089*	
N2	0.52637 (6)	0.39465 (11)	0.67489 (9)	0.0563 (3)	
N3	0.58987 (6)	0.37255 (13)	0.72462 (10)	0.0686 (4)	
H3N	0.6178	0.4232	0.7620	0.082*	
N4	0.02321 (9)	0.7252 (2)	0.03134 (11)	0.0964 (6)	
H4A	0.0003	0.7393	−0.0282	0.116*	
N5	0.09752 (6)	0.57712 (10)	0.31589 (11)	0.0603 (4)	
N6	0.09703 (7)	0.49139 (11)	0.36687 (11)	0.0670 (4)	
H6	0.0749	0.4308	0.3390	0.080*	
O1	0.57234 (6)	0.19418 (10)	0.66782 (9)	0.0769 (4)	
O2	0.66952 (6)	0.27132 (13)	0.76297 (11)	0.0956 (5)	
O1W	0.0000	0.30761 (12)	0.2500	0.0550 (4)	
O3	0.16253 (6)	0.58794 (9)	0.50562 (9)	0.0702 (3)	
O4	0.12715 (6)	0.41067 (10)	0.49677 (10)	0.0790 (4)	
H1WA	−0.0053 (11)	0.2590 (17)	0.2837 (14)	0.110 (7)*	

### Atomic displacement parameters (Å^2^)

**Table:**

	*U*^11^	*U*^22^	*U*^33^	*U*^12^	*U*^13^	*U*^23^
C1	0.0528 (9)	0.0430 (8)	0.0676 (10)	0.0006 (6)	0.0410 (8)	0.0033 (7)
C2	0.0606 (11)	0.0536 (10)	0.0968 (13)	−0.0079 (8)	0.0522 (11)	−0.0047 (9)
C3	0.0542 (11)	0.0768 (13)	0.1139 (16)	−0.0024 (9)	0.0541 (12)	−0.0064 (11)
C4	0.0640 (12)	0.0660 (11)	0.1093 (15)	0.0177 (9)	0.0574 (12)	0.0080 (10)
C5	0.0641 (11)	0.0464 (8)	0.0772 (11)	0.0035 (7)	0.0515 (10)	0.0014 (7)
C6	0.0533 (9)	0.0443 (8)	0.0554 (9)	0.0002 (6)	0.0401 (8)	0.0017 (6)
C7	0.0784 (12)	0.0476 (9)	0.0950 (13)	−0.0127 (8)	0.0659 (11)	−0.0078 (8)
C8	0.0590 (10)	0.0474 (8)	0.0620 (10)	−0.0076 (7)	0.0446 (9)	−0.0042 (7)
C9	0.0545 (10)	0.0576 (10)	0.0632 (10)	−0.0147 (7)	0.0425 (9)	−0.0093 (7)
C10	0.0535 (10)	0.0675 (11)	0.0551 (9)	0.0027 (8)	0.0327 (8)	0.0019 (8)
C11	0.0888 (17)	0.129 (2)	0.133 (2)	0.0305 (15)	0.0641 (16)	−0.0249 (17)
C12	0.123 (2)	0.1079 (18)	0.0932 (16)	0.0552 (16)	0.0886 (17)	0.0451 (14)
C13	0.1119 (19)	0.0849 (15)	0.130 (2)	0.0228 (13)	0.1002 (19)	0.0324 (14)
C14	0.0701 (12)	0.0744 (12)	0.0918 (14)	0.0033 (9)	0.0564 (11)	0.0109 (10)
C15	0.0549 (10)	0.0629 (10)	0.0582 (9)	0.0075 (7)	0.0362 (8)	0.0080 (8)
C16	0.0570 (10)	0.0627 (10)	0.0539 (9)	0.0217 (8)	0.0365 (8)	0.0073 (7)
C17	0.0815 (13)	0.0883 (14)	0.0601 (11)	0.0357 (11)	0.0494 (11)	0.0148 (10)
C18	0.0670 (13)	0.1005 (17)	0.0687 (13)	0.0171 (11)	0.0306 (11)	−0.0243 (12)
C19	0.0501 (9)	0.0642 (11)	0.0613 (10)	0.0093 (8)	0.0304 (9)	−0.0114 (8)
C20	0.0497 (10)	0.0529 (9)	0.0816 (13)	−0.0011 (7)	0.0380 (10)	−0.0145 (9)
C21	0.0554 (10)	0.0384 (8)	0.0911 (13)	−0.0019 (7)	0.0497 (10)	−0.0021 (8)
C22	0.1127 (18)	0.0771 (14)	0.0996 (17)	−0.0153 (12)	0.0692 (16)	0.0080 (11)
N1	0.0903 (11)	0.0386 (7)	0.1144 (12)	0.0056 (7)	0.0758 (11)	0.0035 (7)
N2	0.0464 (8)	0.0592 (8)	0.0643 (8)	−0.0069 (6)	0.0364 (7)	−0.0063 (6)
N3	0.0460 (8)	0.0722 (9)	0.0825 (10)	−0.0108 (7)	0.0394 (8)	−0.0202 (8)
N4	0.0883 (13)	0.1306 (17)	0.0476 (9)	0.0431 (12)	0.0341 (10)	0.0016 (10)
N5	0.0542 (8)	0.0436 (7)	0.0782 (10)	−0.0021 (6)	0.0410 (8)	−0.0039 (6)
N6	0.0647 (9)	0.0428 (7)	0.0899 (11)	−0.0131 (6)	0.0486 (9)	−0.0071 (7)
O1	0.0685 (8)	0.0574 (7)	0.0732 (8)	0.0012 (6)	0.0321 (7)	0.0016 (6)
O2	0.0550 (8)	0.1026 (11)	0.1118 (11)	0.0021 (7)	0.0465 (8)	−0.0286 (9)
O1W	0.0567 (9)	0.0410 (8)	0.0685 (10)	0.000	0.0412 (8)	0.000
O3	0.0713 (8)	0.0426 (6)	0.0865 (9)	−0.0090 (5)	0.0468 (7)	−0.0039 (6)
O4	0.0910 (10)	0.0490 (7)	0.1064 (11)	−0.0161 (6)	0.0684 (9)	−0.0004 (6)

### Geometric parameters (Å, °)

**Table:**

C1—C2	1.367 (2)	C13—H13	0.9300
C1—C6	1.403 (2)	C14—C15	1.372 (2)
C1—H1A	0.9300	C14—H14	0.9300
C2—C3	1.390 (3)	C15—C16	1.399 (2)
C2—H2	0.9300	C15—H15	0.9300
C3—C4	1.368 (3)	C16—C17	1.409 (2)
C3—H3	0.9300	C16—C19	1.442 (2)
C4—C5	1.389 (2)	C17—N4	1.382 (3)
C4—H4	0.9300	C18—N4	1.344 (3)
C5—N1	1.382 (2)	C18—C19	1.371 (3)
C5—C6	1.406 (2)	C18—H18	0.9300
C6—C8	1.441 (2)	C19—C20	1.434 (3)
C7—N1	1.347 (2)	C20—N5	1.272 (2)
C7—C8	1.370 (2)	C20—H20	0.9300
C7—H7	0.9300	C21—O3	1.2055 (18)
C8—C9	1.434 (2)	C21—N6	1.336 (2)
C9—N2	1.285 (2)	C21—O4	1.3502 (19)
C9—H9	0.9300	C22—O4	1.427 (2)
C10—O1	1.2038 (19)	C22—H22A	0.9600
C10—O2	1.331 (2)	C22—H22B	0.9600
C10—N3	1.336 (2)	C22—H22C	0.9600
C11—O2	1.447 (3)	N1—H1	0.8600
C11—H11A	0.9600	N2—N3	1.3868 (18)
C11—H11B	0.9600	N3—H3N	0.8600
C11—H11C	0.9600	N4—H4A	0.8600
C12—C13	1.367 (3)	N5—N6	1.3887 (19)
C12—C17	1.384 (3)	N6—H6	0.8600
C12—H12	0.9300	O1W—H1WA	0.928 (15)
C13—C14	1.398 (3)		
			
C2—C1—C6	118.95 (14)	C13—C14—H14	119.5
C2—C1—H1A	120.5	C14—C15—C16	119.54 (16)
C6—C1—H1A	120.5	C14—C15—H15	120.2
C1—C2—C3	121.17 (16)	C16—C15—H15	120.2
C1—C2—H2	119.4	C15—C16—C17	118.01 (18)
C3—C2—H2	119.4	C15—C16—C19	134.57 (15)
C4—C3—C2	121.77 (17)	C17—C16—C19	107.38 (17)
C4—C3—H3	119.1	N4—C17—C12	130.9 (2)
C2—C3—H3	119.1	N4—C17—C16	106.6 (2)
C3—C4—C5	117.33 (16)	C12—C17—C16	122.4 (2)
C3—C4—H4	121.3	N4—C18—C19	110.9 (2)
C5—C4—H4	121.3	N4—C18—H18	124.6
N1—C5—C4	130.18 (15)	C19—C18—H18	124.6
N1—C5—C6	107.65 (14)	C18—C19—C20	125.10 (19)
C4—C5—C6	122.17 (15)	C18—C19—C16	105.46 (19)
C1—C6—C5	118.61 (14)	C20—C19—C16	129.43 (15)
C1—C6—C8	134.87 (14)	N5—C20—C19	121.37 (15)
C5—C6—C8	106.51 (13)	N5—C20—H20	119.3
N1—C7—C8	110.71 (14)	C19—C20—H20	119.3
N1—C7—H7	124.6	O3—C21—N6	126.17 (16)
C8—C7—H7	124.6	O3—C21—O4	124.73 (18)
C7—C8—C9	122.58 (14)	N6—C21—O4	109.10 (14)
C7—C8—C6	106.16 (14)	O4—C22—H22A	109.5
C9—C8—C6	131.26 (14)	O4—C22—H22B	109.5
N2—C9—C8	124.40 (14)	H22A—C22—H22B	109.5
N2—C9—H9	117.8	O4—C22—H22C	109.5
C8—C9—H9	117.8	H22A—C22—H22C	109.5
O1—C10—O2	124.77 (17)	H22B—C22—H22C	109.5
O1—C10—N3	124.98 (16)	C7—N1—C5	108.96 (13)
O2—C10—N3	110.25 (15)	C7—N1—H1	125.5
O2—C11—H11A	109.5	C5—N1—H1	125.5
O2—C11—H11B	109.5	C9—N2—N3	113.83 (13)
H11A—C11—H11B	109.5	C10—N3—N2	119.82 (14)
O2—C11—H11C	109.5	C10—N3—H3N	120.1
H11A—C11—H11C	109.5	N2—N3—H3N	120.1
H11B—C11—H11C	109.5	C18—N4—C17	109.66 (16)
C13—C12—C17	117.93 (19)	C18—N4—H4A	125.2
C13—C12—H12	121.0	C17—N4—H4A	125.2
C17—C12—H12	121.0	C20—N5—N6	115.05 (14)
C12—C13—C14	121.1 (2)	C21—N6—N5	118.48 (13)
C12—C13—H13	119.4	C21—N6—H6	120.8
C14—C13—H13	119.4	N5—N6—H6	120.8
C15—C14—C13	120.9 (2)	C10—O2—C11	115.48 (17)
C15—C14—H14	119.5	C21—O4—C22	116.13 (15)
			
C6—C1—C2—C3	0.4 (3)	C15—C16—C17—C12	−1.0 (2)
C1—C2—C3—C4	0.2 (3)	C19—C16—C17—C12	−179.00 (16)
C2—C3—C4—C5	−0.2 (3)	N4—C18—C19—C20	177.95 (16)
C3—C4—C5—N1	−179.76 (19)	N4—C18—C19—C16	−1.3 (2)
C3—C4—C5—C6	−0.5 (3)	C15—C16—C19—C18	−176.42 (17)
C2—C1—C6—C5	−1.0 (2)	C17—C16—C19—C18	1.06 (17)
C2—C1—C6—C8	179.73 (16)	C15—C16—C19—C20	4.4 (3)
N1—C5—C6—C1	−179.47 (14)	C17—C16—C19—C20	−178.17 (15)
C4—C5—C6—C1	1.1 (2)	C18—C19—C20—N5	−169.80 (16)
N1—C5—C6—C8	−0.04 (17)	C16—C19—C20—N5	9.3 (3)
C4—C5—C6—C8	−179.45 (17)	C8—C7—N1—C5	0.4 (2)
N1—C7—C8—C9	179.48 (14)	C4—C5—N1—C7	179.13 (19)
N1—C7—C8—C6	−0.42 (19)	C6—C5—N1—C7	−0.2 (2)
C1—C6—C8—C7	179.56 (17)	C8—C9—N2—N3	179.82 (14)
C5—C6—C8—C7	0.28 (17)	O1—C10—N3—N2	−4.1 (3)
C1—C6—C8—C9	−0.3 (3)	O2—C10—N3—N2	175.87 (15)
C5—C6—C8—C9	−179.62 (16)	C9—N2—N3—C10	−163.03 (15)
C7—C8—C9—N2	−175.11 (16)	C19—C18—N4—C17	1.1 (2)
C6—C8—C9—N2	4.8 (3)	C12—C17—N4—C18	178.02 (19)
C17—C12—C13—C14	−1.2 (3)	C16—C17—N4—C18	−0.4 (2)
C12—C13—C14—C15	−0.3 (3)	C19—C20—N5—N6	−178.25 (13)
C13—C14—C15—C16	1.1 (3)	O3—C21—N6—N5	2.6 (2)
C14—C15—C16—C17	−0.5 (2)	O4—C21—N6—N5	−177.00 (13)
C14—C15—C16—C19	176.80 (17)	C20—N5—N6—C21	−177.52 (14)
C13—C12—C17—N4	−176.32 (18)	O1—C10—O2—C11	8.4 (3)
C13—C12—C17—C16	1.8 (3)	N3—C10—O2—C11	−171.5 (2)
C15—C16—C17—N4	177.52 (13)	O3—C21—O4—C22	−4.5 (2)
C19—C16—C17—N4	−0.45 (17)	N6—C21—O4—C22	175.15 (16)

### Hydrogen-bond geometry (Å, °)

**Table:**

Cg4 and Cg5 are the centroids of the C16–C19/N4 and C12–C17 rings, respectively.

**Table:**

*D*—H···*A*	*D*—H	H···*A*	*D*···*A*	*D*—H···*A*
N1—H1···O3^i^	0.86	2.15	2.9426 (18)	154.
O1W—H1WA···N2^ii^	0.93 (2)	2.19 (2)	3.0773 (17)	161.(2)
O1W—H1WA···O1^ii^	0.93 (2)	2.60 (2)	3.2217 (14)	125.(2)
N6—H6···O1W	0.86	2.17	3.0087 (17)	166.
C7—H7···Cg4^iii^	0.93	2.85	3.594 (3)	137
C9—H9···Cg5^iii^	0.93	2.74	3.537 (3)	145
C22—H22A···Cg4^iv^	0.96	2.92	3.555 (4)	125
C22—H22A···Cg5^iv^	0.96	2.86	3.796 (3)	125

Symmetry codes: (i) −*x*+1/2, −*y*+3/2, −*z*+1; (ii) −*x*+1/2, −*y*+1/2, −*z*+1; (iii) −*x*, *y*−1, −*z*+1/2; (iv) −*x*+1/2, *y*+3/2, −*z*+1/2.
